# Thymoma in Myasthenia Gravis: From Diagnosis to Treatment

**DOI:** 10.4061/2011/474512

**Published:** 2011-08-10

**Authors:** Fredrik Romi

**Affiliations:** Department of Neurology, Haukeland University Hospital, 5021 Bergen, Norway

## Abstract

One half of cortical thymoma patients develop myasthenia gravis (MG), while 15% of MG patients have thymomas. MG is a neuromuscular junction disease caused in 85% of the cases by acetylcholine receptor (AChR) antibodies. Titin and ryanodine receptor (RyR) antibodies are found in 95% of thymoma MG and 50% of late-onset MG (MG onset ≥50 years), are associated with severe disease, and may predict thymoma MG outcome. Nonlimb symptom profile at MG onset with bulbar, ocular, neck, and respiratory symptoms should raise the suspicion about the presence of thymoma in MG. The presence of titin and RyR antibodies in an MG patient younger than 60 years strongly suggests a thymoma, while their absence at any age strongly excludes thymoma. Thymoma should be removed surgically. Prethymectomy plasmapheresis/iv-IgG should be considered before thymectomy. The pharmacological treatment does not differ from nonthymoma MG, except for tacrolimus which is an option in difficult thymoma and nonthymoma MG cases with RyR antibodies.

## 1. Thymoma in Myasthenia Gravis

Thymomas in myasthenia gravis (MG) are neoplasms derived from thymic epithelial cells, and are usually of the cortical subtype (WHO type B) [1]. 50% of thymoma patients develop MG (hereafter referred to as thymoma MG in this paper) [2, 3]. Cortical thymomas usually have some morphological similarities with thymic cortex; they share the capacity to propagate the maturation of immature naive CD4 T cells and export mature naive T cells into the periphery. Thymomas lacking this ability do not induce MG [4]. Thymomas with histological similarities to medullary thymic tissue or thymomas lacking developing T cells are seldom associated with MG [4]. Other thymoma characteristics that can cause reduced self-tolerance include defective epithelial expression of the autoimmune regulator (AIRE) gene and/or of major histocompatibility complex class II molecules, absence of myoid cells, failure to generate FOXP3(+) regulatory T cells, and genetic polymorphisms affecting T-cell signalling [5].

Histologically, thymomas are epithelial neoplastic cells surrounded by maturing T cells. The epithelial cells are capable of expressing epitopes cross-reactive with skeletal muscle proteins, such as acetylcholine receptor (AChR), titin, and ryanodine receptor (RyR) [6, 7]. The muscle-like epitopes are presented to T cells together with costimulatory molecules [7]. Autoreactive T cells specific for AChR and titin are found both in thymomas and in thymoma MG patients' sera [8]. Thymoma epithelial cells present AChR peptides to T-cell lines in thymoma MG patients, facilitating intrathymic immunization [9]. 

The patient's genetic profile and the thymic ability to export autoreactive T cells are equally important in developing MG. MG has a genetic association to HLA-DR3 or ancestral haplotype 8.1 in early-onset MG (MG onset before age 50 years) with thymic hyperplasia and several weaker associations to polymorphisms in immunoregulatory genes such as Fc*γ*R, TNF-*α*/*β*, GM-phenotypes, CTLA-4 [10], HLA, and PTPN22∗R620W [11]. The chance of having a thymoma increases with the number of thymoma-associated polymorphisms in an MG patient, indicating that thymoma MG is a polygenic disease and that thymoma patients with a particular genetic profile run higher risk of developing MG [11].

## 2. Thymoma MG

MG is a neuromuscular junction disease characterized by muscular weakness and fatigability, caused in 85% of the cases by AChR antibodies [12]. When MG occurs together with a thymoma, MG is a paraneoplastic disease caused by the presence of the thymoma. Thymoma MG accounts for around 15% of all MG cases [13].

The immune response against an epitope expressed on thymoma cells spills over to neuromuscular junction components sharing the same epitope [14]. In thymoma MG, epitopes are shared between the thymoma and muscle proteins.

## 3. Antibodies in Thymoma MG

AChR antibodies are the main cause of muscle weakness in thymoma MG [15]. Additional non-AChR muscle autoantibodies reacting with striated muscle titin and RyR antigens are found in up to 95% of MG patients with a thymoma and in 50% of late-onset MG patients (MG onset at age of 50 years or later) [16]. These antibodies are usually associated with more severe MG [13, 17–19]. Striational antibodies demonstrated in immunofluorescence are largely made up of titin antibodies [20].

Titin is the largest known protein, with a molecular mass of 3000 kD stretching throughout the sarcomere, providing a direct link between mechanical muscle strain and muscle gene activation [21]. Myositis and myopathy with muscle atrophy are seen in some thymoma MG patients [22]. Sera from MG patients also induce degenerative changes in muscle cell cultures where both apoptosis and necrosis are implicated [23].

The RyR is the calcium channel of the sarcoplasmic reticulum (SR). Upon opening, the RyR releases Ca^2+^ into the sarcoplasm resulting in muscle contraction. In vitro, RyR antibodies can inhibit Ca^2+^ release from the SR [24]. There is also a rat model with thymoma and MG with RyR antibodies but no AChR antibodies, indicating that RyR antibodies may cause MG symptoms irrespective of AChR antibodies [25]. There are also several reports of excitation-contraction coupling defects in thymoma MG [26].

## 4. Recognizing the Clinical and Serological Pattern of Thymoma MG

MG patients with RyR antibodies are characterized by frequent involvement of bulbar, respiratory, and neck muscles at MG onset and a more severe disease. Neck weakness at MG onset is a distinctive feature of patients with RyR antibodies, while respiratory symptoms are also found in patients with titin antibodies with and without RyR antibodies. Limb involvement with few or no bulbar signs is typical at MG onset in RyR-antibody-negative MG [27]. Since many thymoma MG patients have RyR antibodies, neck weakness and nonlimb bulbar distribution of MG symptoms are initial characteristic features associated with thymoma MG. Such symptom distribution should always raise the suspicion about the presence of a thymoma in an MG patient. 

Thymoma MG is equally frequent in males and females and occurs at any age with a peak onset around 50 years [28]. Thymoma MG and late-onset MG share similar serological profile with high prevalence of titin and RyR antibodies and lower AChR antibody concentrations compared to early-onset MG [29]. About 95% and 70% of thymoma MG patients have titin and RyR antibodies, respectively (Table 1). Around 58% and 14% of late onset MG patients have titin and RyR antibodies, respectively (Table 1) [13].

Late MG onset age, similar serological profile, favorable pharmacological treatment response, severe MG, frequent use of immunosuppressive drugs, and the occurrence of MG related mortality are common features among thymoma MG and late-onset MG patients [29]. This profile differs from early-onset MG [30], that has higher AChR antibody concentrations, almost no titin or RyR antibodies, low need for immunosuppressive drugs, less severe MG, very low MG mortality rates, and a favorable thymectomy outcome [29]. 

Thymoma MG tends to be more severe than early-onset nonthymoma MG [29]. In one study, MG patients with thymoma or thymic atrophy (i.e., chiefly late-onset MG) had worse prognosis than MG patients with thymic hyperplasia (i.e., early-onset MG) [31]. The presence of a thymoma per se does not give a more severe MG. Thymoma MG patients and age-matched nonthymoma MG patients share similar MG long-term prognosis [19]. The presence of titin and RyR antibodies is associated with more severe disease in thymoma MG and in late-onset MG [29]. The AChR antibody serum concentration does not correlate with MG severity, mainly because of individual variations in AChR epitope specificity [32].

## 5. Verifying the Diagnosis of Thymoma MG

The diagnosis of MG is based on clinical disease history and typical clinical findings. MG can be confirmed pharmacologically by edrophonium (Tensilon) test which is positive in 90% of MG patients, giving an immediate but transitory improvement of MG signs [33]. The diagnosis of MG should be confirmed by the detection of AChR antibodies, present in most MG cases. These antibodies are present in virtually all patients with a thymoma [29]. In two thirds of MG patients, failure of neuromuscular transmission in leads to decremental response to repetitive nerve stimulation by electromyographical (EMG) examination [34]. Increased jitter on single-fiber EMG is even more sensitive than repetitive nerve stimulation when performed on affected muscles [34]. 

In addition to MG, a thymoma should be demonstrated in order to fulfill the criteria of thymoma MG diagnosis. The diagnosis of a thymoma in MG is finally established by histopathological examination postsurgery. Titin and RyR antibodies and radiological examination of the anterior mediastinum share similar sensitivity for the presence of a thymoma in MG [29, 35, 36]. However, the presence of titin and RyR antibodies in a MG patient younger than 60 years strongly suggests a thymoma, while the absence of such antibodies at any age strongly excludes thymoma [13, 37]. Retesting for these antibodies and a new radiological examination should always be considered whenever clinical deterioration is seen over time, to minimize the risk of a previously undetected thymoma in a MG patient.

## 6. Surgical Treatment of Thymoma MG

When the diagnosis of a thymoma in a MG patient is established, the neoplasm should be removed surgically, and it is crucial to ensure radical excision of the neoplasm. Thymectomy can be performed transternally or through a video-assisted thoracoscopic approach, usually with similar outcome [38]. Radical excision of a thymoma does in most cases cure the thymic neoplasia, but patients will continue to suffer from MG after thymectomy, emphasizing the need of continuing followup and pharmacological treatment. When the thymoma invades the pleura or the pericardium, radical excision will not be possible and further oncological treatment is necessary. Presurgery plasmapheresis or intravenous infusion of immunoglobulin (iv-IgG) removes a great deal of circulating pathogenic antibodies [36]. In our department we give plasmapheresis or iv-IgG treatment to all patients with thymoma MG prior to thymectomy, to minimize the risk of postthymectomy MG exacerbation and myasthenic crisis. This practice varies however from department to another, and there is no consensus on this issue. Iv-IgG should be considered as first choice in patients at high risk of developing cardiopulmonary failure secondary to fluid overload caused by plasmapheresis [39]. MG outcome after thymectomy is generally less favorable in patients older than 45 years (i.e., mostly late-onset and thymoma MG patients) [40].

## 7. Treatment of MG Crisis in Thymoma MG

Plasmapheresis and immunoglobulin treatments are also indicated in severe cases of thymoma MG regardless of thymectomy, such as in MG crisis and in severe MG cases with poor response to standard pharmacological treatment [41]. Parallel to plasmapheresis and immunoglobulin treatment, the pharmacological treatment should be intensified in these patients as explained in the next chapter.

## 8. Pharmacological Treatment of Thymoma MG

The first pharmacological choice in the treatment of thymoma MG is acetylcholinesterase inhibitors. The second choice is immunosuppressive drugs whenever additional pharmacological treatment is needed before or after thymectomy. Several immunosuppressive drugs are available, such as corticosteroids, azathioprine, cyclophosphamide, cyclosporine, methotrexate, mycophenolate mofetil, rituximab, and tacrolimus. Steroids such as prednisolone are frequently given on alternate days, by gradually raising the dose to 60–80 mg initially and then with slowly tapering to 20 mg or lower. If long-term treatment with steroids is regarded necessary, nonsteroid immunosuppressants such as azathioprine should be introduced in addition (usually 100–150 mg a day). While the steroid effect appears rapidly, the clinical effect of other immunosuppressants may take a few weeks to several months to develop [29]. Overall, about 80% of MG patients and 95% of thymoma MG patients need immunosuppressive drug treatment for more than one year [42].

Tacrolimus, which is an immunosuppressant and enhancer of RyR-related sarcoplasmic calcium release, may be especially beneficial in MG patients with RyR antibodies that in theory might block the RyR interfering with its function. Since most patients with thymoma MG have RyR antibodies, tacrolimus may act specifically in these patients. It may have a purely symptomatic effect in addition to its immunosuppressive impact [40]. Tacrolimus has demonstrated favorable effects in the treatment of MG, both as monotherapy and as add-on to prednisolone [43, 44]. Patients should undergo a thorough cardiological investigation prior to commencing tacrolimus treatment. 

Long-term observation of thymoma MG and age-matched nonthymoma MG patients showed no difference in MG severity over time, and both groups improved to the same degree after MG diagnosis as a result of pharmacological treatment and thymectomy. The need for immunosuppressive treatment in the two groups was similarly high. A thymoma that has been completely removed surgically does not necessarily mean worse MG prognosis in thymoma MG [19].

## Figures and Tables

**Table 1:** The occurrence of the various muscle autoantibodies (ab) in the different subgroups of MG [13].

MG subgroup	AChR ab	MuSK ab	Titin ab	RyR ab
Early onset (non MuSK nonthymoma)	Positive in all patients	Negative in all patients	Positive in 10% of the patients	Negative in all patients
Late onset (non-MuSK nonthymoma)	Positive in all patients	Negative in all patients	Positive in 58% of the patients	Positive in 14% of the patients
MuSK positive (regardless onset age)	Negative in all patients	Positive in all patients	No information available	No information available
Seronegative (regardless onset age)	Negative in all patients	Negative in all patients	Negative in all patients	Negative in all patients
Thymoma (regardless of onset age)	Positive in all patients	May occur in some patients	Positive in 95% of the patients	Positive in 70% of the patients
